# Grooming Behavior in American Cockroach is Affected by Novelty and Odor

**DOI:** 10.1155/2014/329514

**Published:** 2014-10-21

**Authors:** Marianna I. Zhukovskaya

**Affiliations:** Sechenov Institute of Evolutionary Physiology and Biochemistry, Russian Academy of Sciences, 44 Thorez Pr., Saint Petersburg 194223, Russia

## Abstract

The main features of grooming behavior are amazingly similar among arthropods and land vertebrates and serve the same needs. A particular pattern of cleaning movements in cockroaches shows cephalo-caudal progression. Grooming sequences become longer after adaptation to the new setting. Novelty related changes in grooming are recognized as a form of displacement behavior. Statistical analysis of behavior revealed that antennal grooming in American cockroach, *Periplaneta americana* L., was significantly enhanced in the presence of odor.

## 1. Introduction

Self-grooming is an innate behavior that is represented across wide range of animal species, such as land vertebrates and arthropods. Despite only far relatedness between these groups, the main features of grooming behavior are amazingly similar and serve the same needs, namely, body cleaning and disease prevention, distribution of some substances across body surface, and as a displacement behavior in stressful conditions [1–3].

Body cleaning is the most obvious purpose of grooming behavior; in insects it can be elicited by small particles, irritants, and weak mechanical stimulation [4–9]. Care of the body surface is thought to be important for disease prevention by elimination of pathogens [10, 11], fungi [12], and also parasites and parasitoids [13, 14]. Along with listed above, grooming is used to spread some substances throughout the integuments [11, 15, 16].

Grooming of various body parts is organized in particular sequences, usually with cephalocaudal progression [2], although no set sequences were found for cockroaches [17]. Frustrated animals perform displacement activities that are identified as locomotory behaviors, cleaning behaviors (e.g., grooming), and manipulation of objects, patterns of which are often disturbed [18–21].

Robust orientation toward the odor source is a vital trait for the insects of various species. Volatiles activating odorant receptors inside olfactory sensilla are eliminated by odor-specific as well as nonspecific enzymes [22, 23], terminating the response. However, odor molecules adsorbed by sensillar and antennal cuticle are likely to travel towards the pores [24, 25] and approach receptor cells long after the odor is gone. According to our recent finding [26] the layer of liquid hydrocarbons which builds up on the surface of antennal flagellum is taken out while passing it through the mouthparts. Hydrophobic odorant molecules get adsorbed and dissolved by the hydrocarbons and should be removed to maintain high temporal resolution of odor signal. Thus, the turnover of antennal lipidous layer serves dual function: directing odorant molecules towards pores on the sensillar walls [27] and removing the odorant from the antennal surface.

Pheromone-degrading enzymes were found on the surface of antennae, wings, and legs of some moth species [28, 29], but clearance of other odors belonging to different classes of chemical substances was not considered. It was noticed that restrained antenna is becoming wetted after a course of odor stimulation and was suggested that liquid exudation is likely functioning in removing residual ligands [30]. Since the liquid is removed by grooming [26], the purpose of current study was to describe in detail the frequency and timing of this particular behavior with special emphasis on novelty and odor traits.

## 2. Materials and Methods

Male last-instar nymphs of* Periplaneta americana* L. were transferred from the stock colony to the cage and were reared under 12 : 12 LD regime at 28 ± 1°C. After the last molt cockroaches were transferred into the experimental setup, they were kept for at least two weeks prior to tests. The experiments started in the first half of a dark phase, a period of maximum locomotor activity [31] and pheromone sensitivity [32]. Water and food were provided ad libitum.

**Figure 1:**
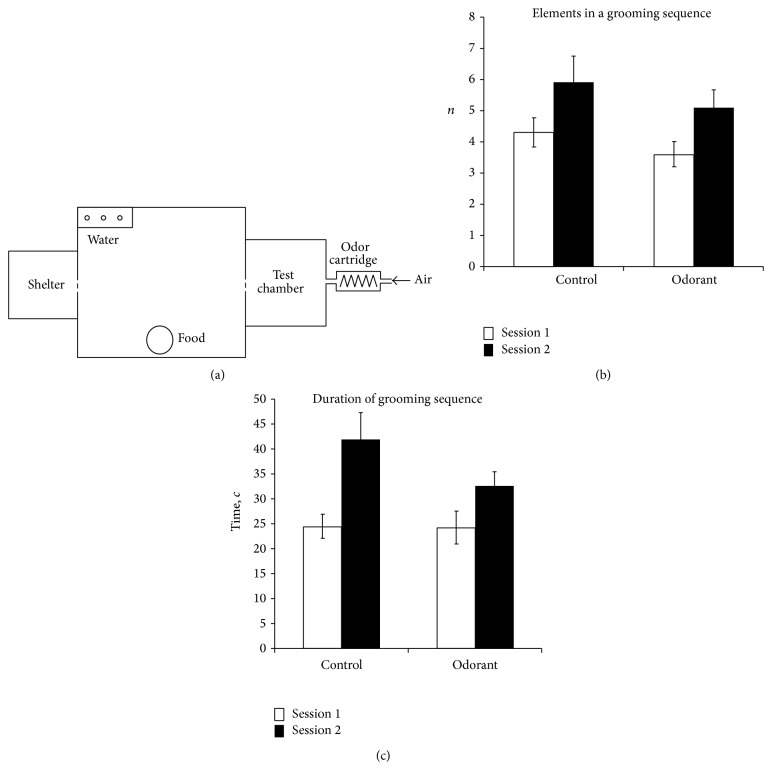
(a) Scheme of experimental setup. (b) The average number of events in a grooming sequence. Error bars are standard errors. (c) Average duration of grooming sequence (s). Error bars are standard errors.

The setup consisted of 3 components: a transparent plastic cage (30 × 45 × 30 cm) containing food and water, a shelter (17.0 × 17.0 × 5.5 cm) that was constantly dark inside, and an exchangeable test chamber (20 × 20 × 8 cm); the latter 2 were separated from the cage with plastic doors (modified from [30]). At the beginning of testing, the door between the shelter and the cage was shut, and the door between the cage and test chamber was open for a short time, allowing only one insect to enter in it. Odor cartridge was loaded with 0.1 mL of 0.1% v/v solution of eucalyptol (1,3,3-trimethyl-2-oxabicyclo[2,2,2]octane, Fluka) in mineral oil (oleum vaselini, P 71.273.2, Tver Pharmaceutical Factory) (Figure 1(a)); 0.1 mL of pure oil was used in control experiments. Artificial air (21% O_2_ and 79% N_2_) was blown through the cartridge with flow rate 3.5 mL/min. Two consecutive video recording sessions lasting 30 min started after a 10 min acclimation period and followed each other with a 10 min break. After an experiment the test chamber was removed and the cockroach was not returned to the cage. This scheme was chosen to avoid marking of the cockroaches, since it was shown to affect grooming in insects [33]. Three series of experiments were conducted. 

*Series 1 (Blank).* Cartridge was loaded by control stimulus in both sessions (*n* = 8). 

*Series 2 (Odor).* Cartridge was loaded by control stimulus in the first session and with eucalyptol in the second session (*n* = 13). 

*Series 3.*  Cartridge was loaded with control stimulus, only one session was run (*n* = 5). For statistical evaluations purposes the data of this series was pooled with those from the first session of Series 1 and Series 2, so the sample size became *n* = 26.

Video files were processed manually frame by frame. Following grooming events were counted and timed separately: cleaning of antennal flagellum by mouthparts; scraping of antennal base by foreleg; cleaning of forelegs and midlegs/hindlegs (pooled together) by mouthparts; scraping of cerci and genitalia by hindlegs. To evaluate locomotor activity, the top-view projection of the test chamber was divided into 4 quadrants and the number of visited quadrants was calculated.

Obtained values were transferred to MS Excel for further data processing and statistical evaluations. Differences (session 2 − session 1) were calculated for every cockroach from each series for each groomed body part (Tables 2 and 3). Two-factor ANOVA with repeated measures on one factor (two consecutive sessions X Series 1 and 2) was used to compare data on the grooming of a particular body part from Series 1 and 2. This procedure was chosen to remove the extraneous variability that derives from preexisting differences in grooming values between series (online calculator, http://vassarstats.net/). Results of these tests are available in Supplementary Table available online at http://dx.doi.org/10.1155/2014/329514. Kolmogorov-Smirnov goodness of fit test (http://contchart.com/goodness-of-fit.aspx) was performed before running Student's *t*-test (for normally distributed data) or Mann-Whitney and Wilcoxon signed-ranks test (in other cases).

## 3. Results

The background grooming frequencies were calculated from the first sessions of Series 1, 2, and 3 (Table 1). Since cerci, sternum, and genitalia were cleaned rarely (we observed just 9 events through 26 tests, which lasted in average 2.1 ± 1.2 s.) they were excluded from further analysis.

Grooming sequences mostly started from antennal grooming followed by scraping antennal bases with ipsilateral foreleg and passing this foreleg through mouthparts and facultatively ended with midleg or/and hindleg grooming. 74.6 ± 4.8% of grooming events was comprised in sequences which lasted 75.0 ± 4.9% of all grooming time.

Preexisting differences between Series 1 and 2 were found for the frequencies in grooming of antennae (*P* < 0.01, Student's *t*-test), antennal bases (*P* < 0.05 Mann-Whitney test), and forelegs (*P* < 0.05, Student's *t*-test), but not midlegs and hindlegs (*P* > 0.05, Mann-Whitney test) as well as for the time spent for grooming (*P* < 0.05 for antennae, antennal bases, and forelegs, Mann-Whitney test) (Table 2). The first look at the differences (session 2 − session 1) in grooming values suggests that these values are dissimilar between Series 1 and 2. Two-way ANOVA with repeated measures on one factor, performed to test the dissimilarities statistically, revealed clear changes in antennal grooming in presence of the odorant, but not in grooming the other body parts; namely, the antennal flagella were cleaned more frequently when the odor was presented (*P* < 0.05) in comparison with control. There was no difference across consecutive sessions for the number of observed flagellum groomings and for the time spent for grooming. Total time spent on antennal maintenance (antennal flagella, antennal bases, and foreleg cleaning taken together) reveals significant influence of the both factors: odor presence (*P* < 0.05) and consecutive sessions (*P* < 0.05). Duration of a single cleaning event of antennal flagellum raised at the second session (*P* < 0.01) and differences were significant for both series (*P* < 0.05, Student's *t*-test); there was no difference between Series 1 and 2 in duration of antennal cleaning events. Antennal bases were groomed more often and took more time in the second sessions (*P* < 0.01), but the odor did not significantly affect this behavior (*P* > 0.05) (Tables 2, 3, and 4). Duration of an antennal base cleaning event was the same for all sessions. Odor did not affect midleg and hindleg grooming, but more frequent (*P* < 0.01) and longer (*P* < 0.05) cleaning events were observed in the second session; also the time spent for grooming increased (*P* < 0.01) (Tables 2, 3, and 4).

Grooming sequences took longer time and contained in average more elements at the second sessions (two-way ANOVA with repeated measures on one factor, *P* < 0.01 for the time and *P* < 0.05 for number of elements) than in the first ones. The changes in sequence parameters look similar for both series (Figure 1), but the longest grooming sequences (41.9 ± 5.3 s) were observed for the second sessions of control series.

Proportion of grooming events comprised in sequences was similar for all samples (two-way ANOVA with Repeated Measures on One Factor, *P* > 0.05), but the small difference between two control consecutive sessions was statistically significant (*P* = 0.02, Wilcoxon signed-ranks test). Sessions of the Series 2 did not differ (*P* > 0.1).

Locomotor activity was not affected by odor (*P* > 0.05, two-way ANOVA with repeated measures on one factor), but uniform decrease was found for both series (*P* < 0.0001, Table 6).

## 4. Discussion

Our data on* P. americana* background grooming obtained for the combined control group are in agreement with those reported earlier [34], namely, the overall time spent for grooming in our experiments was 129.4 ± 16.7 s versus 66 ± 66 s in referred study (the difference was statistically insignificant, Student's *t*-test, *t* = 0.93, *P* > 0.05). Percentage of antennal grooming is also similar: 35% [34], 36% in our preliminary experiments where cockroaches were tested in groups in their living cage [35], and 47% in this study, where animals were tested separately in a clean cage. A slightly higher proportion of antennal grooming in the present study is most likely due to the absence of contaminations which cause cleaning of legs that were in contact with contaminants. Brief analysis of grooming in our experiments questions the postulated lack of particular patterns. The majority of grooming events were organized in sequences (Table 5), 67% of which started with antennal grooming and additional 22% with foreleg grooming. Foreleg was sometimes cleaned immediately before it grasped the flagellum, so this behavior may be related to antennal grooming and viewed as an optional step of the sequence. Although sequences were relatively flexible, a trend to anterior-posterior progression similar with that described for vertebrates [2] was obvious in contrast to that previously reported for cockroaches [17].

Importance of grooming behavior for the maintenance and acuity of sensory organs was suggested for some insects in different circumstances [36–38], but as far as we know was not experimentally examined. Grooming as nonspecific mechanism of signal cleanup is probably a widespread phenomenon; it was noticed in different insect species challenged with pheromone and nonpheromone odors [39–41]. Our present data shows that eucalyptol, the general odorant which causes excitation of a receptor cell housed in male-specific pheromone-sensitive antennal sensilla [30, 42] induces significant changes in the frequency of antennal grooming.

Grooming is a complex multipurpose behavior which is reflected in its “microstructure” [43]. Exposure to novelty is a traditional approach in research of displacement behavior studies in vertebrates and it causes abnormal patterns and interrupted bouts in rodents [43–45]. Shorter grooming sequences displayed by cockroaches in the first sessions (Figures 1(b) and 1(c)) appear to be a form of displacement behavior due to transition to the new surroundings—cleaned box and absence of conspecifics. Shorter cleaning events in the first sessions of both experiments are also in line with well-known stress-related behavioral phenomena [18]. Application of odor in the second session of Series 2 rearranges behavior in favor of antennal grooming. Stressful condition results in multiple changes in biogenic amine levels, which in turn could contribute to the alterations in grooming behavior [46]. Decrease in agitation level could also be recognized by increasing the number of elements in grooming sequences of the second sessions of both series. Locomotor activity data, which shows significant decline in movements with time independently from presence or absence of odor, also support this statement. It is worth emphasizing that odor stimulus did not produce noticeable changes in length and timing of grooming sequences and did not alter duration of either grooming event, so odor presented in our experiments does not cause features of displacement behavior and shows the absence of agitation response. Thus, changes in antennal grooming clearly correlate with odorant perception by an insect.

Importance of chemosensory-dependent grooming should not be overlooked in applied studies, since excessive grooming affects target behavior of beneficial insects [47]. Sugar dusting increased grooming activity decreased bee infestation with* Varroa* mites [9], so application of odors would provide supplemental or alternative treatment.

## 5. Conclusions

Novelty stress disturbs grooming pattern by shortening grooming sequences and duration of cleaning events, similarly with phenomena known for birds and mammals. These data raise a question on origin and evolution of physiological mechanisms involved in manifestations of displacement behavior throughout the main lineages of animal taxa. Males of* P. americana* cockroaches clean more often their main olfactory organs, the antennal flagella, in presence of eucalyptol, the plant odor, underpinning the importance of grooming behavior in olfactory reception.

## Supplementary Material

Two-way ANOVA with Repeated Measures on One Factor was performed for the number of grooming events, time spent for the particular kind of grooming during the whole session, and duration of a single grooming event (Sessions 1-2 as repeated measures and Series 1-2 as independent factors). Online calculator http://vassarstats.net was used to obtain P values.

## Figures and Tables

**Table 1:** Background grooming (averaged over 26 experiments, mean ± standard error).

	Antennae	Antennal bases	Forelegs	Midlegs	Hindlegs
Number of observed events	9.3 ± 1.1	1.7 ± 0.4	6.7 ± 1.0	1.2 ± 0.4	0.3 ± 0.2
Time spent for grooming (s)	69.3 ± 9.0	5.7 ± 1.4	34.2 ± 5.9	14.9 ± 3.8	3.5 ± 1.6

**Table 2:** The number of observed grooming events (count). Mean ± Standard error. Difference was calculated for each animal.

Series	Session	Antennae	Antennal bases	Forelegs	Midlegs and hindlegs	SUM
Series 1 *n* = 8	1 (blank)	14.3 ± 2.2∗∗	2.9 ± 0.4∗	9.3 ± 1.1∗	2.0 ± 0.5	28.4 ± 3.8∗∗
	2 (blank)	10.0 ± 2.3	6.0 ± 1.5	11.8 ± 2.1	3.6 ± 1.1	31.4 ± 5.7
	Difference (2 − 1)	−4.3 ± 1.4	3.1 ± 1.9	2.5 ± 2.0	1.6 ± 1.3	

Series 2 *n* = 13	1 (blank)	7.2 ± 1.3	1 ± 0.5	4.5 ± 1.2	1.5 ± 0.5	14.2 ± 2.9
	2 (odor)	6.8 ± 0.9	3.9 ± 1.1	11.2 ± 1.9	3.2 ± 0.5	25.1 ± 3.5
	Difference (2 − 1)	−0.3 ± 1.2	2.9 ± 1.0	6.6 ± 1.5	1.7 ± 0.4	

Statistical differences between first sessions of Series 1 and 2: ^*^ *P* < 0.05;  ^**^ *P* < 0.01.

**Table 3:** Time spent for grooming (s). Mean ± Standard error. Difference was calculated for each animal.

Series	Session	Antennae	Antennal bases	Forelegs	Midlegs and hindlegs	SUM
Series 1 *n* = 8	1 (blank)	100.4 ± 20.0∗	8.8 ± 2.3∗	43.8 ± 6.9∗	13.1 ± 3.1	166 ± 29.4
	2 (blank)	87.8 ± 23.4	21.9 ± 5.4	66.1 ± 11.3	37.3 ± 11.6	213 ± 43.7
	Difference (2 − 1)	−12.6 ± 18.1	13.1 ± 6.4	22.4 ± 12.4	24.1 ± 11.5	

Series 2 *n* = 13	1 (blank)	54.8 ± 10.8	4.2 ± 2.2	22.2 ± 6.2	13.2 ± 5.4	94.3 ± 21.2
	2 (odor)	57.8 ± 7.3	12.2 ± 3.4	56.3 ± 9.8	31.5 ± 6.4	157.8 ± 20.7
	Difference (2 − 1)	3.1 ± 9.8	8.1 ± 3.0	34.2 ± 9.2	18.2 ± 6.7	

Statistical differences between first sessions of Series 1 and 2: ^*^ *P* < 0.05.

**Table 4:** Duration of a single grooming event (s). Mean ± standard error.

Series	Session	Antennae	Antennal bases	Forelegs	Midlegs and hindlegs
Series 1 *n* = 8	1 (blank)	6.9 ± 0.6	3.0 ± 0.4	4.7 ± 0.4	6.9 ± 1.1
	2 (blank)	8.7 ± 0.6	3.8 ± 0.4	5.9 ± 0.6	10.8 ± 0.9

Series 2 *n* = 13	1 (blank)	7.6 ± 0.5	4.0 ± 1.2	4.5 ± 0.6	8.3 ± 1.7
	2 (odor)	8.7 ± 0.6	3.3 ± 0.4	5.1 ± 0.4	9.9 ± 1.0

**Table 5:** Grooming events comprised in sequences (in percents to all grooming events, mean ± standard error).

	Session 1	Session 2
Series 1 (blank)	76.6 ± 4.9%	87.6 ± 4.6%
Series 2 (odor)	70.8 ± 10.8%	83.5 ± 5.5%

**Table 6:** Locomotor activity (quadrants visited, mean ± standard error).

	Session 1	Session 2
Series 1 (blank)	81.9 ± 8.7	36.0 ± 10.0
Series 2 (odor)	107.8 ± 18.5	51.8 ± 10.0

## References

[1] Borchelt P. L., Denny M. R. (1980). Care of the body surface (COBS). *Comparative Psychology: An Evolutionary Analysis of Animal Behavior*.

[2] Sachs B. D. (1988). The development of grooming and its expression in adult animals. *Annals of the New York Academy of Sciences*.

[3] Hefetz A., Soroker V., Dahbi A., Malherbe M. C., Fresneau D. (2001). The front basitarsal brush in Pachycondyla apicalis and its role in hydrocarbon circulation. *Chemoecology*.

[4] Hlavac T. F. (1975). Grooming systems of insects: structure, mechanics. *Annals of the Entomological Society of America*.

[5] Reingold S. C., Camhi J. M. (1978). Abdominal grooming in the cockroach: development of an adult behavior. *Journal of Insect Physiology*.

[6] Vandervorst P., Ghysen A. (1980). Genetic control of sensory connections in *Drosophila*. *Nature*.

[7] El-Awami I. O., Dent D. R. (1995). The interaction of surface and dust particle size on the pick-up and grooming behaviour of the German cockroach *Blattella germanica*. *Entomologia Experimentalis et Applicata*.

[8] Matheson T. (1997). Hindleg targeting during scratching in the locust. *The Journal of Experimental Biology*.

[9] Stevanovic J., Stanimirovic Z., Lakic N., Djelic N., Radovic I. (2012). Stimulating effect of sugar dusting on honey bee grooming behaviour. *Entomologia Experimentalis et Applicata*.

[10] Kovac D., Maschwitz U. (1989). Secretion-grooming in the water bug *Plea minutissima*: a chemical defence against microorganisms interfering with the hydrofuge properties of the respiratory region. *Ecological Entomology*.

[11] Lusebrink I., Dettner K., Seifert K. (2008). Stenusine, an antimicrobial agent in the rove beetle genus *Stenus* (Coleoptera, Staphylinidae). *Naturwissenschaften*.

[12] Yanagawa A., Shimizu S. (2007). Resistance of the termite, *Coptotermes formosanus* Shiraki to *Metarhizium* anisopliae due to grooming. *BioControl*.

[13] Peng Y.-S., Fang Y., Xu S., Ge L. (1987). The resistance mechanism of the Asian honey bee, *Apis cerana* Fabr., to an ectoparasitic mite, *Varroa jacobsoni* Oudemans. *Journal of Invertebrate Pathology*.

[14] Vincent C. M., Bertram S. M. (2010). Crickets groom to avoid lethal parasitoids. *Animal Behaviour*.

[15] Kovac D., Maschwitz U. (1990). Secretion-grooming in aquatic beetles (Hydradephaga): a chemical protection against contamination of the hydrofuge respiratory region. *Chemoecology*.

[16] Graystock P., Hughes W. O. H. (2011). Disease resistance in a weaver ant, *Polyrhachis dives*, and the role of antibiotic-producing glands. *Behavioral Ecology and Sociobiology*.

[17] Smith B. J. B., Valentine B. D. (1985). Phylogenetic implications of grooming behavior in cockroaches (Insecta: Blattaria). *Psyche*.

[18] Tinbergen N. (1951). *The Study of Instinct*.

[19] Wilz K. J. (1970). The disinhibition interpretation of the “displacement” activities during courtship in the three-spined stickleback, Gasterosteus aculeatus. *Animal Behaviour*.

[20] Anselme P. (2008). Abnormal patterns of displacement activities: a review and reinterpretation. *Behavioural Processes*.

[21] Root-Bernstein M. (2010). Displacement activities during the honeybee transition from waggle dance to foraging. *Animal Behaviour*.

[22] Maïbèche-Coisne M., Nikonov A. A., Ishida Y., Jacquin-Joly E., Leal W. S. (2004). Pheromone anosmia in a scarab beetle induced by in vivo inhibition of a pheromone-degrading enzyme. *Proceedings of the National Academy of Sciences of the United States of America*.

[23] Leal W. S. (2013). Odorant reception in insects: roles of receptors, binding proteins, and degrading enzymes. *Annual Review of Entomology*.

[24] Kanaujia S., Kaissling K. E. (1985). Interactions of pheromone with moth antennae: adsorption, desorption and transport. *Journal of Insect Physiology*.

[25] Maitani M. M., Allara D. L., Park K. C., Lee S. G., Baker T. C. (2010). Moth olfactory trichoid sensilla exhibit nanoscale-level heterogeneity in surface lipid properties. *Arthropod Structure and Development*.

[26] Böröczky K., Wada-Katsumata A., Batchelor D., Zhukovskaya M., Schal C. (2013). Insects groom their antennae to enhance olfactory acuity. *Proceedings of the National Academy of Sciences of the United States of America*.

[27] Böröczky K., Park K. C., Minard R. D., Jones T. H., Baker T. C., Tumlinson J. H. (2008). Differences in cuticular lipid composition of the antennae of *Helicoverpa zea*, *Heliothis virescens*, and *Manduca sexta*. *Journal of Insect Physiology*.

[28] Ferkovich S. M., Oliver J. E., Dillard C. (1982). Pheromone hydrolysis by cuticular and interior esterases of the antennae, legs, and wings of the cabbage looper moth, Trichoplusia ni (Hübner). *Journal of Chemical Ecology*.

[29] Vogt R. G., Riddiford L. M. (1986). Scale esterase: a pheromone-degrading enzyme from scales of silk moth Antheraea polyphemus. *Journal of Chemical Ecology*.

[30] Zhukovskaya M. I. (2012). Modulation by octopamine of olfactory responses to nonpheromone odorants in the cockroach, *Periplaneta americana* L. *Chemical Senses*.

[31] Lipton G. R., Sutherland D. J. (1970). Activity rhythms in the American cockroach, *Periplaneta americana*. *Journal of Insect Physiology*.

[32] Zhukovskaya M. I. (1995). Circadian rhythm of sex pheromone perception in the male American cockroach, *Periplaneta americana* L. *Journal of Insect Physiology*.

[33] De Souza A. R., Ribeiro B., José N., Prezoto F. (2012). Paint marking social wasps: an evaluation of behavioral effects and toxicity. *Entomologia Experimentalis et Applicata*.

[34] Weisel-Eichler A., Haspel G., Libersat F. (1999). Venom of a parasitoid wasp induces prolonged grooming in the cockroach. *Journal of Experimental Biology*.

[35] Zhukovskaya M. I. (2011). Odorant-dependent changes of the antennal surface secretions in the cockroach, *Periplaneta americana*. *Sensornye Systemy*.

[36] Honegger H.-W., Reif H., Müller W. (1979). Sensory mechanisms of eye cleaning behavior in the cricket *Gryllus campestris*. *Journal of Comparative Physiology A*.

[37] Robinson W. H., Wildey K. B. Antennal grooming and movement behavior in the German cockroach, *Blattella germanica* (L.).

[38] Henderson A. E., Hallett R. H., Soroka J. J. (2004). Prefeeding behavior of the crucifer flea beetle, *Phyllotreta cruciferae*, on host and nonhost crucifers. *Journal of Insect Behavior*.

[39] Östrand F., Anderbrant O., Jönsson P. (2000). Behaviour of male pine sawflies, *Neodiprion sertifer*, released downwind from pheromone sources. *Entomologia Experimentalis et Applicata*.

[40] Marinho C. G. S., Della Lucia T. M. C., Guedes R. N. C., Ribeiro M. M. R., Lima E. R. (2005). *β*-eudesmol-induced aggression in the leaf-cutting ant *Atta sexdens rubropilosa*. *Entomologia Experimentalis et Applicata*.

[41] Yanagawa A., Yokohari F., Shimizu S. (2010). Influence of fungal odor on grooming behavior of the termite, *Coptotermes formosanus* Shiraki. *Journal of Insect Science*.

[42] Fujimura K., Yokohari F., Tateda H. (1991). Classification of antennal olfactory receptors of the cockroach, *Periplaneta americana* L. *Zoological Science*.

[43] Smolinsky A. N., Bergner C. L., LaPorte J. L., Kalueff A. V. (2009). Analysis of grooming behavior and its utility in studying animal stress, anxiety, and depression. *Neuromethods*.

[44] File S. E., Mabbutt P. S., Walker J. H. (1988). Comparison of adaptive responses in familiar and novel environments: modulatory factors. *Annals of the New York Academy of Sciences*.

[45] Kalueff A. V., Tuohimaa P. (2004). Grooming analysis algorithm for neurobehavioural stress research. *Brain Research Protocols*.

[46] Fussnecker B. L., Smith B. H., Mustard J. A. (2006). Octopamine and tyramine influence the behavioral profile of locomotor activity in the honey bee (*Apis mellifera*). *Journal of Insect Physiology*.

[47] Gentry G. L., Barbosa P. (2006). Effects of leaf epicuticular wax on the movement, foraging behavior, and attack efficacy of Diaeretiella rapae. *Entomologia Experimentalis et Applicata*.

