# Supplementary material for: Grooming Behavior in American Cockroach is Affected by Novelty and Odor
Source: ScientificWorldJournal. 2014 Oct 21;2014:329514. doi: 10.1155/2014/329514 (PMC4221865; doi:10.1155/2014/329514)
Supplement: Supplementary file 1 — Two-way ANOVA with Repeated Measures on One Factor was performed for the number of grooming events, time spent for the particular kind of grooming during the whole session, and duration of a single grooming event (Sessions 1-2 as repeated measures and Series 1-2 as independent factors). Online calculator http://vassarstats.net was used to obtain P values. [file 329514.f1.doc]

Supplementary Table. Two-way ANOVA with Repeated Measures on One Factor results.

| Factors | Antennae  n/ t(s)/ d(s) | antennal bases  n/ t(s)/ d(s) | forelegs  n/ t(s)/ d(s) | midlegs+hindlegs  n/ t(s)/ d(s) |
| --- | --- | --- | --- | --- |
| Series (1-2) | 0.022*/0.055/0.62 | 0.065/0.07/0.60 | 0.22/0.14/0.12 | 0.51/0.72/0.59 |
| Session (1-2) | 0.06/0.76/0.003** | 0.005**/0.004**/0.59 | 0.0005**/<0.001**/0.24 | 0.006**/0.003**/0.02* |

Data are probability values for the number of observed events (n), overall time spent for the particular kind of grooming during the whole session (t) and duration of a single grooming event (d).

Asterisks mark the levels of statistical significance * P<0.05, ** P<0.01. Raw data are available in tables 2, 3.
